# Image-based cell sorting using focused travelling surface acoustic waves†

**DOI:** 10.1039/d2lc00636g

**Published:** 2023-01-09

**Authors:** Ahmad Ahsan Nawaz, Despina Soteriou, Catherine K. Xu, Ruchi Goswami, Maik Herbig, Jochen Guck, Salvatore Girardo

**Affiliations:** a Max Planck Institute for the Science of Light & Max-Planck-Zentrum für Physik und Medizin Erlangen Germany jochen.guck@mpl.mpg.de salvatore.girardo@mpl.mpg.de; b Department of Chemistry, University of Tokyo Tokyo Japan

## Abstract

Sorting cells is an essential primary step in many biological and clinical applications such as high-throughput drug screening, cancer research and cell transplantation. Cell sorting based on their mechanical properties has long been considered as a promising label-free biomarker that could revolutionize the isolation of cells from heterogeneous populations. Recent advances in microfluidic image-based cell analysis combined with subsequent label-free sorting by on-chip actuators demonstrated the possibility of sorting cells based on their physical properties. However, the high purity of sorting is achieved at the expense of a sorting rate that lags behind the analysis throughput. Furthermore, stable and reliable system operation is an important feature in enabling the sorting of small cell fractions from a concentrated heterogeneous population. Here, we present a label-free cell sorting method, based on the use of focused travelling surface acoustic wave (FTSAW) in combination with real-time deformability cytometry (RT-DC). We demonstrate the flexibility and applicability of the method by sorting distinct blood cell types, cell lines and particles based on different physical parameters. Finally, we present a new strategy to sort cells based on their mechanical properties. Our system enables the sorting of up to 400 particles per s. Sorting is therefore possible at high cell concentrations (up to 36 million per ml) while retaining high purity (>92%) for cells with diverse sizes and mechanical properties moving in a highly viscous buffer. Sorting of small cell fraction from a heterogeneous population prepared by processing of small sample volume (10 μl) is also possible and here demonstrated by the 667-fold enrichment of white blood cells (WBCs) from raw diluted whole blood in a continuous 10-hour sorting experiment. The real-time analysis of multiple parameters together with the high sensitivity and high-throughput of our method thus enables new biological and therapeutic applications in the future.

## Abstract 2

Real-time morpho-rheological analysis of cells by RT-DC, combined with the improved sorting performance of an on chip FTSAW-based microactuator enables efficient label-free image-based sorting of various cell types with distinct physical properties.

## Graphical abstract

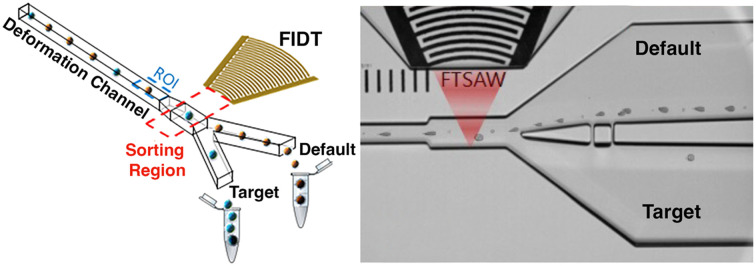

## Introduction

The isolation of specific cells from a heterogeneous population with high sensitivity, reliability and purity, is a fundamental requirement in many biological and clinical research areas such as high-throughput drug screening,^1^ cancer research,^2,3^ rare cell enrichment^4^ and cell transplantation.^5,6^ Different technologies are available to enable cell isolation and can generally be classified as bulk separation methods or single cell sorters. Common bulk cell separation methods include centrifugation,^7^ filtration,^8^ magnetic activated cell sorting (MACS),^9,10^ and microfluidic approaches^11^ such as deterministic lateral displacement (DLD).^12^ Such methods rely on a specific parameter (*e.g.* cell size, deformation, fluorescence) which must be hard-wired into the experimental setup. This means that the selection of a single cell from a heterogeneous population using a combination of parameters is highly challenging with such approaches. Single-cell sorters, though slower in comparison to bulk separation methods, are able to select single cells based on their real-time analysis and sort them into the desired outlets, based on multiple parameters.

Among single cell sorters, the gold standard is fluorescence activated cell sorting (FACS).^13^ FACS is presently a state-of-the-art commercially available product, however, the procedure is time consuming, costly, and requires trained personnel. It relies on the fluorescent labelling of cells, rendering the user blind to cells without known molecular markers. This limits the discovery of new cell populations, for which markers are not yet identified. Moreover, for certain applications, the presence of the fluorescent labels in sorted cells may not be suitable,^14^ such as in personalized medication and transplantation. Furthermore, although forward and side scatter are label-free, they do not provide a direct representation of cell size, granularity or refractive index and hence can be difficult to relate to physical properties of cells.^15^

In recent decades, the label-free characterisation of cells has shown a relationship between inherent cell physical properties and their specific state and function. This has led to the development of novel methods combining microscopy and microfluidics, which perform spatial and temporal analysis of several cell physical and chemical properties with high throughput and sensitivity. Among such methods are Raman scattering,^16^ quantitative phase imaging,^17,18^ impedance- and dielectrophoresis-based analyses,^19,20^ Brillouin microscopy,^21^ suspended micro-channel resonators,^22^ deformability cytometry (DC),^23^ quantitative DC^24^ and real-time deformability cytometry (RT-DC).^25,26^ The further integration of microactuators enabled the sorting of single cells based on the selection and combination of specific parameters, today frequently performed by the use of artificial intelligence (AI).^15,27,28^ This opened a new avenue for label-free cell sorting approaches, such as: dielectrophoresis,^29^ pneumatic valves,^16,27^ on-chip solenoid valve controllers,^30^ optical switches^31^ and pulsed lasers.^32^ However, cell viability,^32,33^ on-chip integration^27^ and throughput^34^ still limit their broader use. Conversely, surface acoustic wave (SAW)-based actuators are compact and easily integrable with microfluidic systems providing high speed actuation without affecting cell viability and functionality.^15,35,36^ Acoustophoretic motion induced by SAW has been widely investigated both experimentally and theoretically,^37^ demonstrating that acoustofluidic-based microfluidic approaches are capable of manipulating cells and particles. Cells or particles can be separated, sorted and focused based on their density, and compressibility, without restrictions based on their electric, magnetic, and optical properties.^38^

Recently, we demonstrated sorting from heterogeneous populations, with >90% purity at rates of up to 100 cells per s, by integrating real-time fluorescence deformability cytometry (RT-FDC) with standing surface acoustic wave (SSAW) actuation.^15^ Cells were imaged in real-time, while they were hydrodynamically aligned and deformed in a microfluidic constriction channel slightly bigger than the cell diameter.^25^ Additionally, our image-based cell sorting approach only requires small sample volumes (∼10 μl), a feature which is critical for rare or precious samples (*e.g.* retina cells, neonatal blood^28,39^). Following the real-time analysis, upon SSAW actuation the cell of interest was transferred to the pressure node aligned in front of the target outlet. Proper operation of the system relied on having one single pressure node precisely located within the microfluidic channel. This limited the maximum IDT frequency and consequently the magnitude of the acoustic force. Furthermore, precise alignment between the IDT and the channel was required, or else further phase adjustments were needed, making the chip fabrication and usage highly challenging and necessitating specialized users. All these aspects restricted the maximum cell concentration, the velocity, and the minimum pulse time, resulting in a maximum throughput of 100 cells per s.

A promising alternative for SSAW is traveling surface acoustic waves (TSAWs). The latter provides more flexibility in IDT design and makes the approach more user-friendly, since it does not require pressure node allocation. Furthermore, the cell displacement distance is not limited by the pressure node position as in the case of SSAW and can be increased by increasing the resonance frequency.^30–32,35^ This generates higher forces, and therefore a shorter pulse time is required for particle displacement compared to SSAW. Furthermore, using a curved, rather than straight, IDT can provide more concentrated energy within a smaller sorting region. Consequently, the cell concentration can be increased and, in combination with a shorter pulse time, can enable higher sorting throughput.

Here, we design and integrate a focused interdigitated transducer (FIDT) with the existing image-based RT-DC microfluidic analysis platform to enable cell sorting at a throughput of up to 400 cells per s. Utilizing 28 μm wavelength FIDTs, FTSAW produces concentrated acoustic energy within a 50 μm region such that a 1 ms pulse is enough to translate the target cell into the desired outlet. This allows one to increase the cell concentration compared to the previous SSAW design.^15^ Furthermore, the new geometry of the RT-DC chip reduces cell clumping and thereby prevents clogging. This enables long sorting experiments, which are fundamental for picking up lowly populated cells within a concentrated heterogeneous population. As a proof of concept, we tested the FIDT-based sorting RT-DC platform (soRT-DC) with various cell types and particles of different physical parameters. We demonstrated high-precision size-based sorting of microgel beads and cell mixtures. We performed deformation and size-based sorting, as well as brightness and size-based sorting, of different white blood cells from RBC-depleted blood with a purity exceeding 92%. The system was able to provide consistent results with increasing cell concentration (up to 36 × 10^6^ cells per ml) without affecting purity (>92%) and cell viability (>90%). Sorting of small entities in the size range of platelets was also possible. We demonstrated, for the first time, a label-free 667-fold enrichment of WBCs from raw diluted whole blood in a continuous 10-hour sorting experiment, opening new avenues for rare-cell sorting. Finally, we showed sorting of sub-populations of cells with different mechanical properties from a homogenous cell population. Our setup provides a user-friendly, label-free, reliable, flexible and high-throughput continuous cell sorting technology that opens new possibilities for both basic biological and therapeutic research. The FTSAW actuator itself, thanks to its flexibility, robustness, easy operation and integration, represents a promising option for sorting integration for forthcoming technologies. In the future, an FIDT with a smaller aperture and higher/multiple frequencies can be developed to further increase the rate of cell sorting.

## Results and discussion

### Sorting principle: FTSAW *vs.* SSAW

**Fig. 1:**
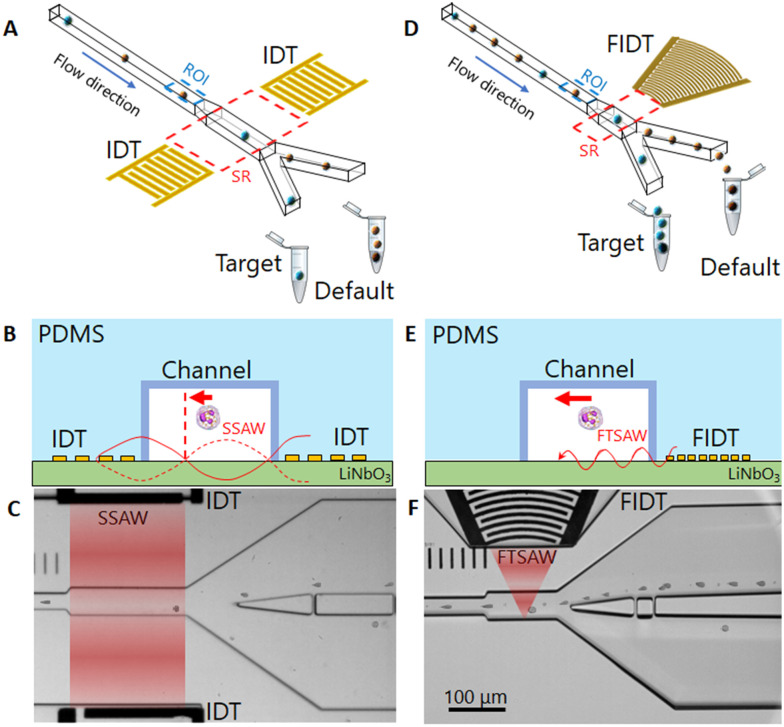
Operation principle of a SSAW and FTSAW-based soRT-DC device. (A) and (D) Hydrodynamically focused cells are deformed in the primary deformation channel and analyzed in real-time in the region of interest (ROI, dotted blue). A cell in the sorting region (SR, dotted red), is pushed towards the target outlet, due to its interaction with (A) SSAW and (D) FTSAW. (B) and (E) Side view of the device cross-section in the sorting region including representation of the surface acoustic wave (red lines) and main force (red arrow) generated by (B) SSAW and (E) FTSAW. For the SSAW, (B) cells are pushed toward the pressure node (vertical dashed red line). For the FTSAW, (E) the cells are pushed away from the FIDT. (C) and (F) Bright-field images of the sorting chips showing the SR during the sorting of WBCs from whole diluted blood for the SSAW and FTSAW, respectively. The red zones indicate the active SR. The concentration of cells is approximately four times higher in FTSAW as compared to SSAW due to smaller and focused SR (red zones).

The sorting principle of SSAW and FTSAW-based soRT-DC devices is illustrated in Fig. 1(A–C) and (D–F), respectively. In the SSAW based sorting approach, two IDTs are placed on opposite sides of the primary channel as indicated in Fig. 1A. The resonance frequency of the IDT is given by 
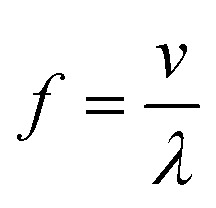
, where *v* is the velocity of sound in LiNbO_3_ (3978 m s^−1^) and *λ* is the acoustic wavelength equal to the periodicity of the fingers (2*d*; *d* is the distance between two consecutive fingers). The primary channel widens from 20 or 30 μm (region of interest, ROI) to 50 μm (sorting region-SR; Fig. 1A and C) to accommodate cell lateral displacement for sorting. The downstream bifurcation is designed so that the default outlet is offset by 5 μm from the center of the primary channel. When the SSAW is off, a hydrodynamically focused particle flows through the main channel into the default outlet. Upon actuation, the constructive interference of counter propagating waves generated by the IDT pairs results in SSAW. By tuning the phase of the sound waves, the pressure node is aligned in front of the target outlet (Fig. 1B). Under such conditions, the acoustic radiation force (ARF), *F*_SSAW_ exerted on the particles is given by^40^1
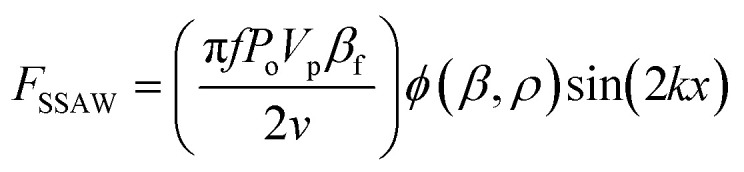
where *P*_o_ is the acoustic pressure, *V*_p_ is the volume of the particle, *β* is the particle compressibility, *ρ* is the particle density, *k* is the acoustic wave number, *x* is the distance between the particle and the pressure node, and *ϕ* is the acoustic contrast factor that describes if the particle will translate to the pressure node (*ϕ* > 0) or the pressure antinode (*ϕ* < 0).2
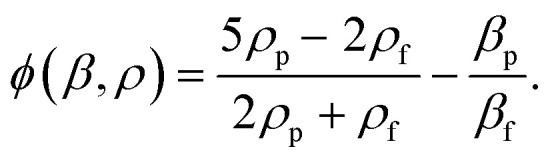
The subscripts p and f stand for particle and fluid, respectively. For a specific particle and fluid, the key parameters which describe sorting with SSAW are: the IDT wavelength (*λ*) and the acoustic pressure (*P*_o_), that is inversely related to the aperture of the IDT (length of overlapping fingers of IDT-see Fig. 1C).^40^ These can be adjusted to allocate a pressure node within the channel in front of the target outlet and provide enough ARF to transfer the cell to it. At the same time, a cell's residence time in the sorting region must be large enough to enable this transfer. Ideally, only one cell should be in the sorting region upon SSAW activation to achieve high sorting purity. All these aspects have been taken into account in the development of our previous sorting method. A resonance frequency of 55 MHz, (defined by *λ* = 70 μm), an IDT aperture of 200 μm equal to the length of the sorting region, and a pulse time of 2 ms were necessary to effectively translate the target cell into the target outlet, limiting the sorting throughput to 100 cells per s at a maximum cell concentration of 1 × 10^6^ cells per ml.

Increasing the throughput can be achieved by increasing the cell concentration, however this requires reductions in the IDT aperture size and pulse time to prevent the sorting of non-target cells along with cells of interest. This in turn necessitates a higher ARF by increasing frequency, which would result in multiple pressure nodes within the channel cross-section. Furthermore, the pressure node setting step makes the sorting system operation tedious. These factors limit the upgradability of the SSAW based sorting approach and hence demand a different strategy to get sorting efficiency close to the RT-DC analysis rate (1000 cell per s *circa*) in a more user-friendly and reliable manner.

TSAW is a good alternative that provides flexibility in device design. TSAW produced by a single IDT has been shown to efficiently actuate fluids for various applications such as mixing^41^ pumping^42^ and nebulization.^43^ Furthermore, TSAW has also been employed in the manipulation of micro-objects for separation,^44^ trapping^45^ and sorting.^46^ The interaction of TSAW with particles in fluids can be classified in two regimes depending on the value of the *κ* factor:

3 *κ* = 2π*d*_p_*f*/*C*_f_

where *d*_p_ is the particle diameter, *f* is the resonance frequency of the IDT and *C*_f_ is the speed of sound in the host medium.^47^ Theoretically, for *κ* > 1, anisotropic wave scattering occurs.^48^ In this regime, ARF is dominant and the time-averaged ARF is described by the following equation:^49^

4 〈*F*_ARF_〉 = *Y*_T_π*d*_p_^2^〈*E*〉/4

where *Y*_T_ is the acoustic radiation factor which is dependent on particle mechanical properties and size^47,50^ and 〈*E*〉 is the time-averaged energy density. The ARF therefore increases with increased particle size.^47^ For *κ* < 1, isotropic wave scattering occurs and acoustic streaming flow (ASF), originating from the dissipation of acoustic waves in the fluid, dominates.^47^ However, experimentally, distinction of the two regimes at *κ* = 1.28 ± 0.2 has also been suggested.^48^ In this regime, ASF induces a drag force on the particle given by *F*_D_ = 3π*ηdv* where *η* is the dynamic viscosity of the medium and *v* is the cell velocity.

The velocity of sound in the measurement buffer (MB) used in our experiments was 1487 m s^−1^ as determined by Brillouin scattering.^21^ Therefore, for our system (*f* = 135 MHz) a *κ* factor (eqn (3)) of 1.28 corresponds to a particle diameter of 2.25 μm. Hence, lateral displacement perpendicular to the flow direction is activated predominantly by ARF for particles larger than 2.25 μm and by ASF for particles smaller than 2.25 μm.

Furthermore, particle displacement due to ARF does not require pressure node setting. This provides more flexibility in the design of IDTs, as frequency selection is not linked to the channel dimensions. IDTs with smaller periodicity can be used to provide higher frequency forces acting on particles in a shorter pulse time. Furthermore, analogous to a convex lens to focus light, a FIDT consisting of concentric circular segmented fingers can focus the acoustic energy within a tight region, thereby reducing the size of the sorting region (Fig. 1D and F).

### soRT-DC chip design

The FIDT employed for the present study is composed of 30 pairs of curved fingers with a periodicity of 28 μm (Fig. S1†) resulting in a resonance frequency of 135 MHz. The FIDTs were designed with an aperture of 100 μm at the front end and 600 μm at the distal end subtending an angle of 42° at the focal point. The focal length of the IDT is 170 μm (Fig. S1†). A single FIDT was placed next to the primary channel, as shown in Fig. 1D. The FIDT was aligned so that the distance between the first FIDT finger and the farther channel wall was equal to the focal length (Fig. S1†). This provided an acoustic energy concentrated within 50 μm in the center of the sorting region (Fig. 1F and S1†). To prevent energy loss, the distal end of the FIDT has reflectors that direct the SAW energy towards the frontal end (Fig. S1†). The design of the FIDT allows a TSAW actuation after real-time cell analysis with a pulse time of 1 ms, half the length of the pulse time required for SSAW.^15^ The FIDT is connected to a 2-channel radio frequency (RF) function generator with an inbuilt power amplifier and can provide a maximum power of 33 dBm (∼2.0 W). For the SSAW, 2 channels were used with a total power of ∼3.8 W, while for FTSAW only a single channel is needed with a power of ∼1.9 W. Theoretically, the generated time-averaged acoustic radiation force 〈ARF〉 scales with *d*_p_^2^ and is dependent on the acoustic radiation factor (*Y*_T_) which is a function of the cell/particle's mechanical properties and size.^47^ In a previous study which employed an FIDT with a resonance frequency of 132 MHz, it was shown that the sorting of 7 μm diameter polystyrene (PS) particles was possible by using a power of 8 mW and a pulse time of 0.25 ms. A higher power (45 mW) and pulse time (1 ms) were necessary to enable an effective displacement of ∼20 μm MCF-7 cells, which although bigger than PS particles, have a lower acoustic contrast factor.^50^ However, the extent of cell displacement is also affected by the viscosity of the buffer; cell displacement may be drastically reduced in a buffer with high viscosity due to the high drag force acting on cells during their movement. For our system, higher power is needed to yield a significant displacement of cells moving in a highly viscous MB.

The new channel geometry has a shorter sorting region of 130 μm, instead of 200 μm used in SSAW sorting region (Fig. 1A and D) and a pillar cascade architecture at the inlet regions, to prevent chip clogging (Fig. S2†). In the sample inlet region, the pillars are followed by six serpentine-like channels, that help to separate cell aggregates (Fig. S2†).^28^ The sheath fluid (MB) and sample fluid (cell suspension) are supplied to the respective inlets *via* syringe pumps while the default outlet is connected to a negative pressure controller (Video S1†). The improved geometry and the use of a negative pressure controller, instead of a syringe pump, as was previously used,^15^ ensure fast flow stabilization and thus enable reliable and long sorting experiments.

### Assembly, interface and operation of soRT-DC chip

The soRT-DC chips presented are used for real-time label-free analysis of cells based on their morpho-rheological properties and downstream sorting of target cells by using FTSAW with a pulse time of 1 ms and a power of 1.9 W. The schematic and the picture of the soRT-DC chip are shown in Fig. 1A and S3.† The design of the FIDT and chip geometry are reported in Fig. S1 and S2.† The soRT-DC chip is composed of two layers: i) the bottom layer is a LiNbO_3_ substrate with a microfabricated FIDT for FTSAW excitation coated with a layer of 100 nm SiO_2_ to enhance the bonding with the polydimethylsiloxane (PDMS) element. The IDT was fabricated by photolithography, E-beam evaporation, subsequent lift-off and RF sputter coating and has a resonance frequency of around 135 MHz, measured with a vector network analyzer ii) the upper layer is a polydimethylsiloxane (PDMS) microstructured element including the microchannel geometry, fabricated by soft lithography. The master templates used to fabricate the PDMS replicas were fabricated by photolithography. The bonding between the two layers was generated by an air plasma treatment. Two different chips have been used for the sorting experiments of cells with different sizes. The chips are characterized by a flow-focusing geometry where cells are focused in a narrow square channel with a side length of 20 μm or 30 μm (deformation channel), which is slightly bigger than the cell diameter to enable cell deformation by hydrodynamic forces in a contact-free manner. The primary deformation channel is followed by a wider (50 μm), 130 μm-long sorting channel, which bifurcates into the default and target channels. The FIDT is aligned in front of the sorting channel and located in a pocket separated from the sorting channel by an 80 μm thick PDMS block. The FIDT design and location provide a focal point located at the sorting channel wall opposite the FIDT.

The assembled device (Fig. S3A†) is mounted on the microscope stage by using a custom-made chip holder which includes the SubMiniature version A (SMA) connectors for its interface with the function generator (Fig. S3B†). It is connected to the external flow controllers *via* fluorinated ethylene propylene (FEP) tubing fitted into the PDMS access holes. The sample and sheath inlets are connected to two syringe pumps, the default outlet to a negative pressure controller and the target outlet to a collection tube. Initially, the chip is flushed with MB through the tubing connected to the sheath inlet until the chip is filled with buffer and all air bubbles are removed. The sample tubing containing the cells or particles is then connected and the sample flow (0.01 μl s^−1^) and sheath flow (0.03 μl s^−1^ and 0.07 μl s^−1^, respectively, for the chip with 20 μm and 30 μm deformation channels) are activated. The flow in the default channel is further adjusted *via* a negative pressure controller to keep the cell stream as close as possible to the bifurcation wall (Video S1†). This is necessary to translate the cell of interest into the target outlet upon FTSAW actuation. After the flow stabilization, the positioning of the ROI within the deformation channel is adjusted to encompass 1 ms delay between cell detection and FTSAW initiation. The sorting parameters are fed into the sorting software based on the sorting gating strategies (Table 1) and the experiment is started momentarily to verify whether the cells can be effectively translated towards the target outlet or if further adjustments in negative pressure and/or ROI positioning are necessary. Before starting the sorting experiment the cell physical properties of interest are analyzed by RT-DC and the resulting scatter plot is used for gating target cells. Based on the gating strategy the initial purity is calculated as the percentage ratio of the number of target cells (cells in the gating area of the scatter plot) to the total number of detected cells. Once the sorting experiment begins, the flowing cells are analyzed in real-time in the principal deformation channel and the feedback system actuates the FIDT to generate FTSAW to translate the target cells into the target outlet. After sorting the cells collected in the target tube are reanalysed by RT-DC and the target purity is calculated as the ratio of the number of cells inside the gating area to the total number of detected cells.

**Table 1:** Experimental details of sorting gating strategies and sorting performance for cells of different sizes and Young's modulus (YM). The apparent Young's modulus and standard deviation (S.D.) were calculated by Shape-Out 2.8.11 software using a look-up table derived from simulations based on the finite element method and an analytical model.^76,77^ The same calculation was not possible for platelets due to their small size and consecutive low resulting deformation. As reference, a YM value from Lam *et al.* is reported^81^

Sample	Size [μm^2^]	Deformation	Brightness	Area ratio	YM ± S.D. [kPa]	IP/TP [%]	Enrichment [fold]
PAAm beads (Fig. 3B)	115–135	—	—	<1.05	1.8 ± 0.9	52.6/86.3	1.64
PAAm beads (Fig. 3C)	125–135	—	—	<1.05	2.1 ± 1.0	26.8/87.7	3.39
Myeloid (Fig. 4A)	53–120	<0.15	—	<1.08	0.8 ± 0.2	36.0/96.7	2.68
Lymphocytes (Fig. 4B)	25–45	<0.1	—	<1.08	1.3 ± 0.8	18.0/92.0	5.11
Neutrophils (Fig. 4C)	56–100	—	100–115	<1.08	1.2 ± 0.4	30.8/96.7	3.13
Platelets (Fig. 4D)	<20		80–100	<1.08	∼10 (ref. 81)	18.0/40.0	2.22
WBC (Fig. 5)	23–100	<0.1	—	<1.08	1.5 ± 1.0	0.09/60.0	667
HL60/S4 (Fig. S6†)	75–125	<0.2	—	<1.08	0.7 ± 0.3	40.1/97.0	2.41
KC167 (Fig. S4A†)	25–77	<0.15	—	<1.08	1.1 ± 0.6	24.1/94.1	4
HL60/S4 (Fig. S4B†)	100–200	<0.15	—	<1.08	0.61 ± 0.07	59.4/91.5	1.54

### Acoustic streaming and cell displacement in soRT-DC chip

**Fig. 2:**
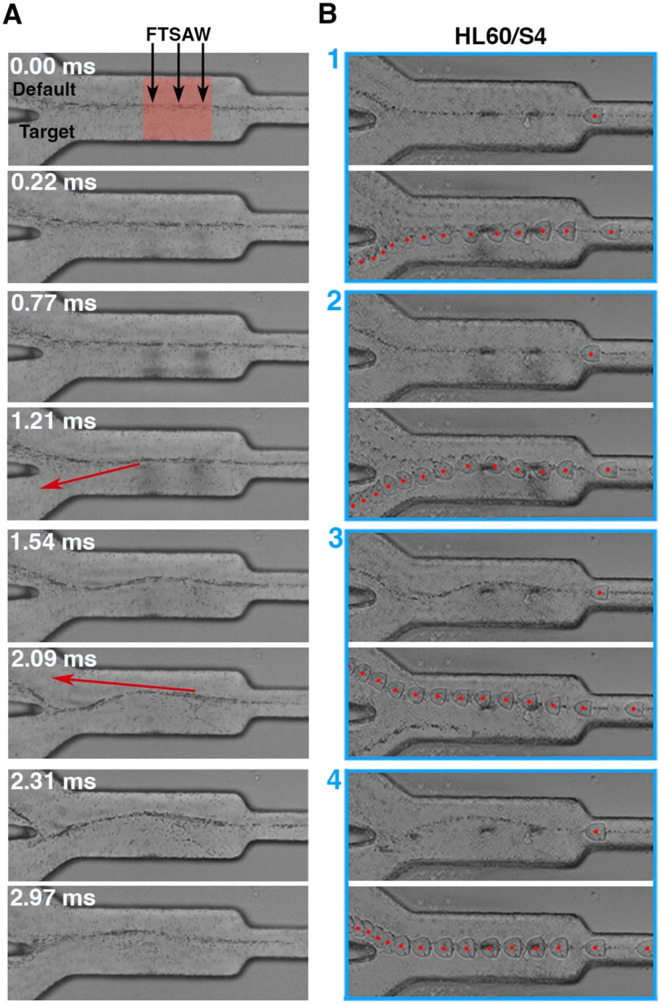
Acoustic streaming and cell displacement. (A) Time evolution of the flow-focused trajectory of 0.6 μm polystyrene particles upon FTSAW actuation with a pulse time of 1 ms. The particles in a 0.01 μl s^−1^ flow are focused in the center of the square deformation channel (size 20 μm) by a focusing 0.03 μl s^−1^ sheath flow. The black arrows show the direction of FTSAW propagation with an energy concentration within a region of 50 μm in the sorting region (SR; red-shaded area). The red arrows show the tangent to the curve in the points where ASF affects the cell trajectory inducing a cell movement towards the target or default channel, as shown in the corresponding frame in the right column. The frames were extracted from a video acquired at 9000 fps and the corresponding time is reported for each image. (B) Brightfield images of HL60/S4 and 0.6 μm PS particles dispersed in MB under the same experimental condition reported for (A). In each panel (blue frame) the top image shows the streamline status at the moment that the cell enters the SR, which is equivalent to the cycle stage shown in the left column. The bottom image in each panel shows the overlay of consecutive frames with a time difference of 0.33 ms to depict the overall cell trajectories. The four panels provide an overview of the acoustic radiation force (ARF) and acoustic streaming flow (ASF) contribution to the cell trajectories. Panel 1: the cell is pushed down by ARF and monotonically moves toward the target outlet. Panel 2: at the entrance of the SR the cell is initially pushed down by ARF, then moves up by ASF, following the distorted streamline until the end of the sorting region where it starts moving toward the target outlet in the direction tangent to the curved streamline (red arrow in the 1.21 ms frame). Panel 3: cell trajectory is affected by ASF. The cell follows the curved streamline in the first half of the sorting channel and continues to move towards the default outlet in the direction tangent to the curved streamline (red arrow in the 2.09 ms frame). Panel 4: cells are not affected by ARF and ASF and move directly towards the default outlet.

The cells in the soRT-DC chip flow in a high viscous MB which is necessary to enhance cell deformation and also ensure slower cell sedimentation within the connecting tubing. The MB is a solution of methylcellulose in PBS (0.6% w/w) and has a shear thinning behaviour with an effective viscosity in the sorting channel of ∼9.8 mPa s and ∼10.1 mPa s, respectively, for the sorting chip with 20 μm (total flow rate 0.04 μl s^−1^) and 30 μm (total flow rate 0.08 μl s^−1^) deformation channels.^51^ The microfluidic chip is characterized by a 2D flow-focusing geometry where cells are focused in the center of the deformation and sorting channels. Cell positioning can vary along the channel height direction due to different cell sizes. The straight sorting channel develops into a downstream bifurcation, where the default outlet is 5 μm offset from the center of the primary channel. When the FTSAW is off, cells flow through the main channel into the default outlet. The default outlet is connected to a negative pressure controller, where the applied negative pressure is adjusted to ensure that all the cells move close to the bifurcation wall and into the default channel when the FTSAW is inactive (Video S1†). Upon actuation, the propagating waves generated by FIDT result in FTSAW, which can displace cells/particles by ARF and/or ASF. Cells moving in MB are subjected to high drag forces; high power is therefore required (∼1.9 W) to produce an effective cell displacement by FTSAW with a pulse time of 1 ms activated by using FIDT with a resonance frequency of 135 MHz. To investigate how the FTSAW activation affects cell migration and MB flow, we performed experiments where the flow-focused trajectory of 0.6 μm polystyrene (PS) particles with and without HL60/S4 cells, was analyzed through the acquisition of high frame rate videos (9000 fps) during 1 ms pulsed focused FTSAW activation at 100 ms intervals. In the time window of 1 ms upon FTSAW actuation, the PS particles continue to move straight in the sorting channel following the laminar flow-focused profile (Fig. 2A). For our system, the minimum particle size for effective ARF particle displacement is around 2.25 μm (*k* > 1). Therefore, for 0.6 μm PS particles the ARF can be neglected and the flow is primarily dominated by the flow drag, here enhanced by the high viscosity of the MB. After 1 ms, ASF perturbed the flow-focused trajectory and the central flow-focused streamline started to develop into a sinusoidal-like wave profile which after 2 ms *circa* disrupts in front of the channel bifurcation (Fig. 2A). The dispersion of HL60/S4 cells together with PS particles provided a clear picture of the impact of ARF and ASF on particle trajectory. In the first 1 ms, HL60/S4 cells, much bigger than PS particles, can be pushed by ARF towards the target outlet (Fig. 2B, panel 1). Interestingly, our analysis shows that in the timeframe of 1 ms a contribution to the cell sorting can be also provided by ASF (Fig. 2B, panel 2). The ASF can still affect the cell trajectory after 1 ms, enhancing the cell displacement towards the default outlet (Fig. 2B, panel 3). After 2 ms, the cells move towards the default outlet, with no observed effects from either ARF or ASF (Fig. 2B, panel 4). To the best of our knowledge, sorting by FTSAW in a highly viscous buffer has not been investigated before and has mostly demonstrated in buffers with viscosity similar to water by using FIDT providing a resonance frequency in the range of 132–386 MHz, an acoustic power between 45 mW to 100 mW, and a pulse time in the range of (25 μs–1 ms).^46,50,52^

### Testing soRT-DC performance

**Fig. 3:**
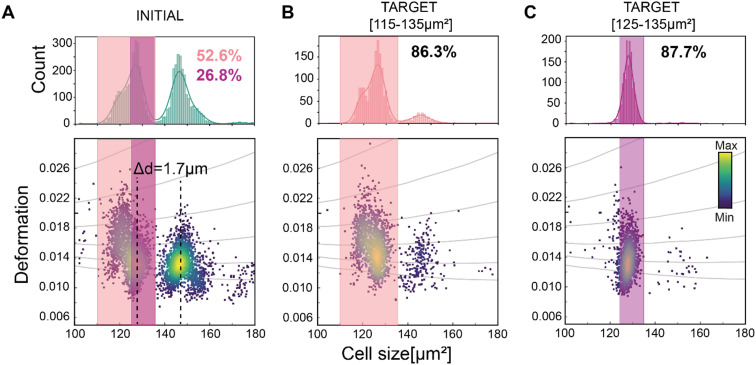
Sorting for narrowing-size distribution of microgel beads: size-based sorting sensitivity. Size-based sorting of a mixture of microgel beads of two different sizes (modal diameter difference, Δ*d* = 1.7 μm). Deformation *vs.* cell size scatter plot of (A) the initial bead populations. The pink and purple rectangles indicate the employed sorting gates: 115–135 μm^2^ and 125–135 μm^2^ respectively. The percentages indicate the number of beads within the sorting gates, 52.6% and 26.8% respectively. (B) Deformation *vs.* bead size scatter plot of the first sorted target population (115–135 μm^2^) showing an 86.3% target purity and of (C) the second target population (125–135 μm^2^) showing an 87.7% target purity. The histograms of bead size presented in the scatter plots are shown on top of the corresponding scatter plots. The histograms were fit with a Gaussian kernel function (solid lines). The color map in the scatter plots represents the event density.

First, the performance of soRT-DC system was tested on polyacrylamide (PAAm) microgel beads that mimic physical properties of cells such as size and elasticity.^53^ We demonstrated precise size-based sorting from a mixture of closely-sized bead populations (with a modal diameter difference, Δ*d*, equal to 1.7 μm), as illustrated in Fig. 3A. We first sorted the smaller bead population using a size range of 115–135 μm^2^ (Fig. 3A). Post-sorting analysis of the target sample showed an increase in purity from 52.6% (initial purity) to 86.3% (target purity) which corresponds to a 1.64-fold enrichment (Fig. 3B). Here, we define target purity (TP) as the percentage of the total number of cells in the sorted sample with the desired properties for sorting, and initial purity (IP) as the percentage of the total unsorted initial sample with the target properties. Enrichment is defined as the ratio of purity in the sorted sample to initial purity. To test the sensitivity of our system, a narrower size sorting gate (125–135 μm^2^; Fig. 3C) was applied within the smaller bead population (with an initial coefficient of variation, C.V. = 3.2%). Analysis of the sorted sample showed a 3.39-fold enrichment, from 26.8% IP to 87.7% TP, with the C.V. reduced to 2.1%.

Size-based sorting was further tested on a cell mixture (Kc167 and HL60/S4 cells mixed in a 1 : 4 ratio), gating for the Kc167 (target gate 25–77 μm^2^, Fig. S4A†), where smaller Kc167 cells can result in a lower acoustic radiation force.^47,54^ A 4-fold enrichment of Kc167 cells was achieved from 24.1% IP to 94.1% TP (Fig. S4A†). Sorting was similarly successful for a mixture of suspension and adherent cells (HL60/S4 and HeLa cells, mixed in 1 : 1 ratio) with a 1.54-fold enrichment from 59.4% IP to 91.5% TP (Fig. S4B†). Our results demonstrate the sensitivity, efficiency and flexibility of our method, as applied to different types of cells and particles. Furthermore, post-sorting analysis of HL60/S4 cells exposed to TSAW showed that the high frequency and acoustic energy concentration within 50 μm did not affect cell proliferation or viability (Fig. S5A and B†). For comparison, we included the following controls: 1) initial: cells which were resuspended in MB but not passed through the microfluidic channel; 2) default: cells collected during the sorting experiment in the default outlet, *i.e.* all non-target cells; 3) culture medium: cells that were never exposed to MB; 4) no SAW: cells that were resuspended in MB and passed through the microfluidic chip without activating the FTSAW. Cell proliferation was monitored by culturing the cells over a 4-day period and assessing their growth and viability daily. As shown in Fig. S5A and B,† exposing HL60/S4 cells to FTSAW (‘target’) or MB buffer (‘initial’) did not affect cell growth or viability, which was comparable to cells that were never exposed to sorting or sorting buffer labelled as ‘culture medium’.

Considering a pulse time of 1 ms, theoretically, approximately 1000 cells could be sorted in a second. However, only one cell must be present in the sorting region to avoid sorting of non-target cells. This limits the maximum sorting rate, given by the product of the maximum cell concentration and the applied flow rate. Provided that the volume of the sorting region is 0.2 nl, the maximum cell concentration in the device is 5 × 10^6^ cells per ml. We applied a total flow rate of 0.08 μl s^−1^ to have a cell residence time (*ca.* 2.5 ms) in the sorting region higher than the pulse time (1 ms). At this flow rate the maximum sorting rate is 400 cells per s. Using a 7 : 1 sheath to sample flow ratio, our system can handle a maximum cell concentration of approximately 40 × 10^6^ cells per ml. To test this, we performed a series of sorting experiments with increasing cell concentration per ml of MB. We tested a range of concentration, starting from 3 × 10^6^ cells per ml up to 36 × 10^6^ cells per ml, and achieved a target purity of >92% (Fig. S5C and S6†) at all cell concentrations. This result demonstrates the robustness of our method across different experimental conditions. Moreover, the average cell recovery for these experiments was approximately (72 ± 15)%. Recovery is defined here as the percentage ratio of the cells collected in the target tube to the total number of cells reported to have been sorted by the instrument. As expected, we observed that the percentage purity of the target cells did not decrease even at the highest maximum concentration allowed by our system.

### Blood cell sorting: parameter selection strategy, small cell size and small cell fraction

Sorting a single cell population from a heterogeneous cell mixture is essential to classify a cell sorter as accommodating for demanding biological applications. Blood is an ideal test-case sample to showcase the flexibility and applicability of our method, as it is a natural heterogeneous cell mixture, consisting of numerous sub-populations of cells.

Blood is a crucial bodily fluid which provides health status of various organs when analysed thoroughly. Traditionally, various diseases are diagnosed only after fluorescent labelling of blood cells and their subsequent analysis *via* FACS.^13^ Deeper investigation may require downstream isolation of diseased, fluorescently labelled blood cells. However, besides adding cost and preparation time, fluorescent labels might alter cells and their functionality. Additionally, the fluorescently labelled sorted cells might not be compatible with downstream applications such as transplantation. Starting from whole blood, RT-DC can be used as a label-free tool to identify all major cell types^25^ and also to detect their pathological changes in several disease states by single-cell morpho-rheological phenotyping of blood.^55,56^ Recently, it was shown that physical phenotype of blood cells is altered in COVID-19 patients' blood.^57^ This might be a unique way for monitoring long-term disease progression in a label-free manner. The sorting of cells based on their morpho-rheological phenotype could play a key role in identifying new biological markers connected to cell physical properties in relation to their functionality. In the future, the analysis and combination of several cell markers at the single-cell level in cooperation with single-cell sorting and single-cell genomics technologies^58^ can be a disruptive method for cell classification in diagnostic applications and also for the discovery of new types of cells.

**Fig. 4:**
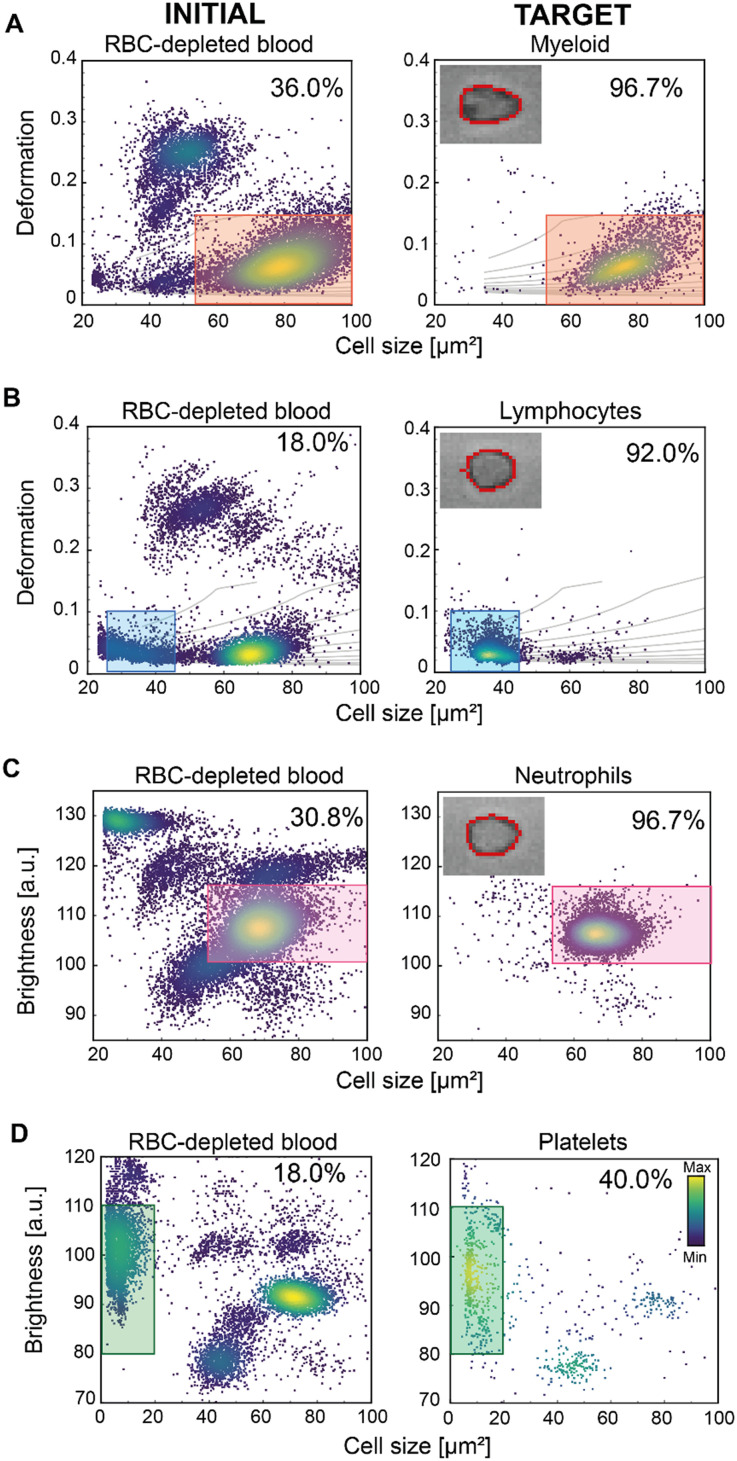
Size, deformation and brightness-based sorting of cells from RBC-depleted blood. (A and B) Deformation *vs.* cell size scatter plots of RBC-depleted blood (dextran-sedimentation). The scatter plots on the left show the initial cell populations, the ones on the right the sorted target cell populations. The insets in the right scatter plots are representative bright-field images including the contour detection (red line) acquired during the RT-DC analysis for (A) a myeloid cell, (B) a lymphocyte, (C) a neutrophil. The gates used for sorting are indicated in orange for myeloid cells (A) and blue for lymphocytes (B). Deformation and cell size-based sorting for myeloid cells with a 2.6-fold enrichment from 36% initial purity to 96.7% target purity (A) and for lymphocytes with a 5-fold enrichment from 18% initial purity to 92% target purity (B). The grey lines in the graphs are isoelasticity lines. (C and D) Brightness and cell size-based sorting of (C) neutrophils and (D) platelets from RBC-depleted whole blood. The gates used for sorting are indicated in pink for neutrophils and green for platelets. The brightness is presented in arbitrary unit (a.u.). The scatter plots on the left represents the initial cell populations and, on the right, the sorted target cell populations. 3.1-Fold enrichment was obtained for neutrophils (from 30.8% to 96.7%); 2.2-fold enrichment for platelets (from 18.0% to 40.0%). The color map in the scatter plots represents the event density. The percentages indicate the number of cells within the sorting gates.

By utilising deformation and cell size alone, three blood cell populations can be distinguished as shown in Fig. 4A: red blood cells, lymphocytes, and granulo-monocytes (GM – often referred to as myeloid cells; myl). We used these parameters to test the ability of soRT-DC to sort myeloid cells and lymphocytes from RBC-depleted (dextran-sedimented) blood. We performed a label-free sorting of myeloid cells based on size (50–100 μm^2^) and deformation (<0.12), achieving a 2.6-fold enrichment from 36.0% IP to 96.7% TP (Fig. 3A). We also sorted lymphocytes based on a size gate of (23–45 μm^2^) and deformation (<0.1), reaching a 5-fold enrichment from 18.0% IP to 92.0% TP (Fig. 4B).

Another strategy to differentiate sub-populations of cells which are otherwise obscure in cell deformation and size analysis is using average brightness and size. This allows the further classification of myeloid cells into neutrophils, monocytes, basophils and eosinophils.^15,56^ We therefore used the average cell brightness together with cell size to assess whether we could successfully sort neutrophils from RBC-depleted blood (Fig. 4C). Using a cell size gate of 50–100 μm^2^ and an average brightness of 100–115 arbitrary units (a.u.), we achieved a 3.1-fold enrichment; an increase in purity from an initial 30.8% to a 96.7% in the sorted sample (Fig. 4C). This proves the high sensitivity and efficiency of our system, at high sorting rates.

Altogether these results show the flexibility of our sorting method based on the combination of multiple physical parameters and the efficacy of the ARF in pushing cells with size bigger than 23 μm^2^ (*κ* > 1, eqn (3)) and with Young's modulus varying in the range of 0.4–3.0 kPa *circa* (Table 1), where both size and cell mechanical properties can affect the cell acoustic contrast factor and consequently the cell displacement by ARF.^47,50^ To test whether, despite the lower ARF, sorting of even smaller cells would be possible, starting from RBC-depleted blood, we used a gating strategy that identifies platelets, which are approximately 2–4 μm in diameter. In this size range, most of the cells can still be pushed by ARF, but due to the force scaling with *d*_p_^2^, the resultant force might be too low to generate an effective displacement towards the target outlet of all analyzed platelets, given the high viscosity of the buffer. Using a gate based on size <20 μm^2^ and brightness of 80–110 a.u., we achieved a 2.2-fold enrichment, from 18.0% IP to 40.0% TP (Fig. 4D). Although the final 40.0% purity is low compared to sorting results obtained for bigger particles, it is the first time that sorting of small cells by TSAW has been shown. In the future, this result might be improved by using FIDT with a higher frequency and smaller aperture. This will open the opportunity to sort even smaller entities with high purity. In a previous study, it was shown that morphorheological characterization of blood can be used to distinguish between viral and bacterial infection.^56^ As sorting of RBCs has already been demonstrated previously^15^ and here improved and extended to still smaller entities, in the future, parasite-infected RBCs or even small living organisms, such as bacteria, could be separated from whole blood. The further downstream analysis would facilitate their genetic identification to determine the most effective antibiotics. Currently, the sorting of smaller entities by TSAW has been mostly demonstrated on polystyrene (PS) particles, having an acoustic contrast factor, *Y*_T_, higher than that of cells.^50^ Ma *et al.* showed sorting of 7 μm PS particles suspended in PBS by using an FIDT with a resonance frequency of 132 MHz operated with a power of 8 mW and a pulse time of 0.25 ms.^50^ They showed that the displacement of ∼20 μm MCF-7 cells by ARF is drastically reduced compared to 7 μm PS particles and higher power (45 mW) and a longer pulse time of 1 ms was necessary to enable their sorting.^50^ Collins *et al.* demonstrated the displacement of smaller 2 μm polystyrene particles under flow in water by using an FIDT with a higher resonance frequency (386 MHz), a higher power of 100 mW and a pulse time of 1 ms.^46^ In our system, cells move in a high viscosity MB, increasing drag force, and thereby requiring higher power to cause their effective displacement towards the target outlet. Nevertheless, we have shown high sorting performance with a purity >92% among several cell types with a wide range of sizes. A significant reduction in sorting performance was observed only for the platelets, at the extreme lower end of the theoretical size range. In addition to the lower ARF, the low sorting efficiency of platelets could be also affected by an increased variation in the particle velocities through the device. Our system employs 2D flow-focusing in the *xy*-plane, so that cells with sizes much smaller than the size of the channel can flow in different *z*-planes with different velocities. Analysing the cell flow velocity in real time and thus adjusting the timing of FTSAW activation could therefore provide a strategy by which to increase the efficiency of sorting small particles. Several methods such as image-based, laser detection, and impedance methods can be used to extract cell velocity within microfluidic channels.^59,60^

**Fig. 5:**
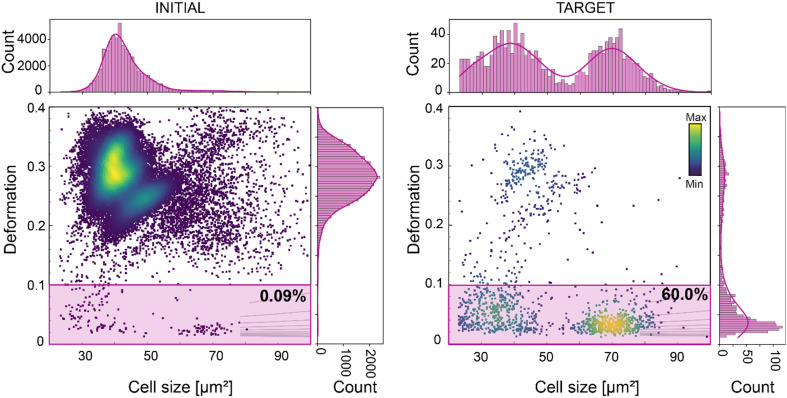
Deformation-based sorting of white blood cells (WBC) from diluted whole blood. Deformation *vs.* cell size scatter plot of whole blood (left graph) and of the sorted cell populations (right graph). The gates used for sorting are indicated in pink. The percentage indicates the number of cells within the sorting gates. The initial percentage of WBC in diluted whole blood was 0.09%. After a 10-hour sorting experiment WBC were enriched to 60%, corresponding to a 667-fold enrichment (right scatter plot). The color map in the scatter plots represents event density. The histograms of features presented in the scatter plots are shown on top and on the right of the corresponding scatter plots. The histograms were fitted with a Gaussian kernel function (solid lines).

Along with the flexibility in sorting strategy, supported by an effective, pure, and high-throughput FTSAW sorting, the stability of the system over time is a fundamental requirement to enable the sorting of small cell fraction within a concentrated heterogeneous population. Here, we demonstrate for the first time the sorting of WBCs from whole diluted blood (1 : 50 dilution in the MB), in a continuous 10-hour long experiment. WBCs comprise only 0.09% of the entire blood population (Fig. 5). The sorting of WBCs based on size (23–100 μm^2^) and deformation (<0.1), resulted in a 667-fold enrichment, yielding a TP of 60% (Fig. 5). This result is not trivial, considering that cells with time tend to cluster and that unprocessed raw blood contains several substances that can lead to chip clogging. With this experiment we showed that the new chip design can support a reliable long sorting experiment, avoiding any channel blockage, due to the new pillar geometry and the six-serpentine channels included after the sample inlet (Fig. S2†). These served to block large-sized debris or cellular aggregates and to break cell clusters, which are often observed during continuous long sorting experiments. During this experiment cells were detected at a rate of ∼400 cells per s, ending with a total of *circa* 14 million screened cells, and a total of *circa* 12 000 sorted WBCs. This demonstrates that our method is applicable for downstream – omics experiments and even cell transplantation experiments, where tens to hundreds of thousands of cells are required.^28,61^ The cell sorting yield can further be enhanced just by increasing the cell sample concentration. We have shown that our system enables sorting at higher cell concentration (up to 36 × 10^6^ cells per ml) while retaining high purity (>92%), (Fig. S5C and S6†) and RT-DC can support cell analysis with a rate of up to 1000 cell per s.

In the future the combination of our sorting platform with deep neural networks (DNNs) can help with the identification and selection of specialized cells and with the discovery of novel cell populations. Different neural networks models have been developed to classify cells. Dendritic cells have been counted reliably *via* image processing algorithms (CasDC) for cancer treatment monitoring,^14^ macrophages,^62^ B and T lymphocytes,^27^ stem cells^63^ and circulating tumor cells^64^ have been also identified based on label-free image-based analysis. DNN-based sorting has been demonstrated in our first paper^15^ and more recently improved and extended to single cells from nervous tissue.^28^ This was achieved by using sorting based on SSAW and the sorting performance can now be improved thanks to FTSAW.

Furthermore, in a recent study, it was shown that a mechanomics approach can be employed to identify the genes involved in the regulation of cell mechanical properties.^65^ The additional single-cell sorting based on cell mechanical properties can open new avenues in mechanobiology of how intrinsic or induced variation in cell mechanical properties can dictate cell function, which could in turn be harnessed for novel diagnostic and therapeutic approaches.^66^

### Sorting based on cell mechanical properties

**Fig. 6:**
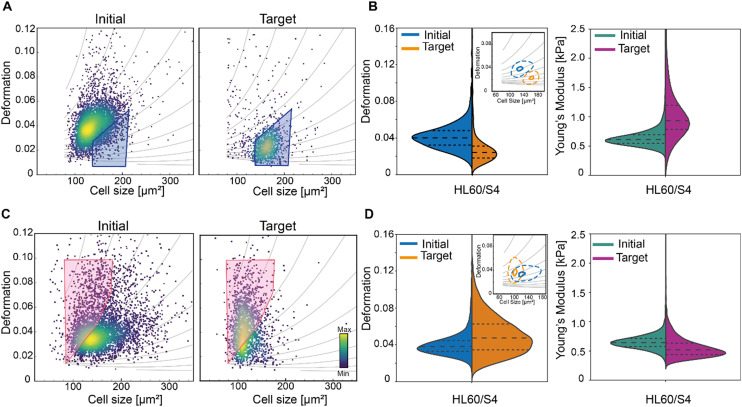
Mechanical properties-based sorting of HL60/S4 cells. (A) and (C) Deformation *vs.* cell size scatter plot of the initial cell populations (left graphs) and of the sorted target cell populations (right graphs). The grey isoelasticity lines on the scatter plot identify cells with comparable mechanical properties over a range of cell sizes. A polygon gate was used to select a sorting region within the isoelasticity lines. The gate used for (A) sorting stiffer cells is indicated in blue and (C) for sorting more compliant cells in pink. The color map in the scatter plots represents the event density. (B) and (D) Violin plots showing the distribution of deformation and Young's moduli for the initial and sorted target cell populations of the corresponding cells in (A) and (C) respectively. The insets show contour plots (dashed lines: 95% density; solid lines 50% density) of deformation *versus* cell size. Young's modulus was calculated using an established theoretical framework.^76,77^

Mechanical properties of cells have been shown to be linked to their functional state,^66^ and are a promising label-free biomarker for cell sorting. Microfluidic cell sorting based on these properties have been demonstrated using both passive and active approaches (summarised in a recent review).^67^ Passive approaches include among others deterministic lateral displacement (DLD) to sort skeletal progenitor cells^68^ or filtration methods to sort leucocytes^8^ or cancer cells^69^ based on their deformation with high throughput. The applicability of such methods is limited to cells with specific physical properties which dictate the microfluidic chip geometry and the flow conditions, that cannot be adjusted during the experiment. Furthermore, information on individual particle deformability cannot be extracted and inherent clogging occurrence with time can compromise the chips functionality and the selectivity of the methods.^67^ These limitations can be overcome by active sorting, providing more flexibility, stability and sensitivity, albeit at the expense of the sorting rate. The first active approach involved the use of a microfluidic optical stretcher to sort single cells based on their mechanical properties.^70^ However, the sorting throughput was limited to 100 cells per h. Dielectrophoresis has been used as an actuation force for cell manipulation,^71^ separation,^72^ trapping^73^ and deformation.^74^ Based on their intrinsic electric properties, cells can be deformed by using dielectrophoresis and a model has been recently introduced to determine a lumped parameter indicative of cells' electrical and mechanical properties. The authors proposed that this parameter can be used in a high throughput system to distinguish between different cell types, but this has not yet been demonstrated.^20^ Other active approaches have been developed to increase the sorting rate, but they showed sorting mostly on a heterogeneous mixture of cells^8,19^ or beads^75^ with distinct mechanical properties. Recently, using a SSAW actuator we demonstrated deformation-based sorting of RBCs.^15^ However, deformation is not solely dependent on mechanical properties but also on cell size, as a bigger cell will experience more stress and thus deform more in the channel.^25,76^ To isolate cells with similar mechanical properties we employed a polygon gate strategy, where a specific cell population (stiff or soft) is selected based on the isoelasticity lines of the deformation-cell size scatter plot. The isoelasticity lines (grey lines in Fig. 6) which are derived from numerical simulations^76,77^ define regions of identical stiffness.^25,76^ We made use of the isoelasticity lines to select a subpopulation of HL60/S4 cells and sorted for either stiffer (Fig. 6A and B) or more compliant (Fig. 6C and D) cells. Re-analysis of the target cells showed an enrichment of the cells within the sorting region (Fig. 6A and C). The Young's modulus distribution of the respective target cells suggested an enrichment for stiffer cells (Fig. 6B) and more compliant (Fig. 6D). The apparent Young's was calculated, using an established theoretical framework.^76,77^ Here, we demonstrated a new gating strategy to separate a homogenous cell population into sub-populations with different mechanical properties. In the future, this can be exploited to sort cells with different mechanical properties and to assess how their functional state varies with stiffness.

## Materials and methods

### FTSAW based soRT-DC chip fabrication

The soRT-DC device substrate is a 128° *Y*-cut lithium niobate (LiNbO_3_). The sodalime chrome photomask (JD Photo Data, U.K.) was designed using Klayout software (version 0.26.8). AZ 5214E photoresist (MicroChemical GmbH, Germany) was spun onto a LiNbO_3_ wafer in a two steps process. Step 1: 500 rpm (100 rpm s^−1^) for 10 s; step 2: 4000 rpm (300 rpm s^−1^) for 30 s. The substrate was soft baked at 112 °C for 60 s followed by UV exposure (at 100 mJ m^−2^) through the photomask *via* a Mask Aligner (MA6 Gen4; Suess MicroTec GmbH). The exposed substrate was developed for 45 seconds using AZ400K developer (MicroChemical GmbH, Germany) diluted 1 : 3 ratio in deionized water to remove the unexposed photoresist. After development, MEB550S E-beam evaporator (PLASSYS, France) was used to deposit on the substrate a layer of 10 nm chromium (Cr), inter-adhesion layer, followed by a layer of 100 nm gold (Au). The photoresist coated with Cr/Au was removed by a lift-off process in acetone. The final substrate has on his surface Cr/Au features reproducing the FIDT geometry. Finally, the substrate was coated with 100 nm of SiO_2_ layer using RF sputter coating (Orion-5, AJA Int. USA). The SiO_2_ layer increases the shelf-life and reusability of FIDT substrates and enhances the bonding strength between the polydimethylsiloxane (PDMS) element and the LiNbO_3_ substrate. The textured PDMS element was generated starting from a master template produced by standard photolithography process. AZ® 15 nXT photoresist (MicroChemical GmbH, Germany) was spun onto a 4-inch SiO_2_ wafer (2000 rpm (5000 rpm s^−1^) for 4.6 s) and soft-baked at 110 °C for 4 minutes. The chip design reported on a photomask was transferred on the coated substrate by UV exposure at 690 mJ m^−2^ (MA6 Gen4; Suess MicroTec GmbH, Germany), followed by a post-baking at 120 °C for 120 s and 150 s development in AZ® 400 K developer, diluted 1 : 3 in deionized water. The final master template was coated with 1*H*,1*H*,2*H*,2*H*-perfluorooctyl-trichlorosilane (no. 448931, Sigma-Aldrich) by vapor deposition under vacuum. This provided a hydrophobic coating for an easy peel-off of the PDMS element during the replica molding process. A PDMS mixture (base to curing agent ratio of 10 : 1 (w/w); no. 634165S, SYLGARD 184, VWR) was poured over the master, degassed, and cured at 78 °C for 1 hour. Harris Uni-Core biopsy punchers (1.5 mm) was used to punch holes for sample/sheath inlets and default/target outlets.

For the chip assembly, LiNbO_3_ substrate and PDMS element were subjected to plasma activation (50 W, 15 s; Plasma Cleaner Atto; Diener Electronic) and assembled after their alignment under a stereo microscope (Zeiss Stemi DV4). The bonding was further strengthened by curing the assembled chip in an oven at 78 °C for 24 hours. Two microfluidic chips were produced with 20 μm or 30 μm squared primary deformation channel.

### Experimental setup and feedback system

The experimental setup consists of an RT-DC system (Zellmechanik Dresden, Germany) interfaced with a radio frequency (RF) function generator (BSG F20, BelektroniG) for FTSAW actuation. The function generator has an inbuilt power amplifier with the capability of producing 2 Watts at each of the two channels. For TSAW only one channel was utilized. FIDT were characterized using a NanoVNA (handheld Vector Network Analyzer: range 50 kHz–900 MHz). It indicated a return loss (*S*_11_) of −10 to −14 dB at the resonance frequency of 135 MHz. The soRT-DC chip was placed onto an inverted microscope (Axio Observer Z1, Zeiss) equipped with a pulsed, high-power LED and an objective (Plan-Apochromat, 20×/0.8; no. 440640-9903, Zeiss). Birefringence of LiNbO_3_ was corrected using a polarizer (Polarizer D, 90° rotatable, removable; no. 427706-0000-000, Zeiss). Image acquisition was performed at 2700 fps by a high-speed CMOS camera (EoSens CL, MC1362, Mikrotron), linked to a standard PC *via* frame grabber card (PCIe-1433, National Instruments).

A custom C++ based software, developed on an OpenCV platform, was used for the image acquisition and processing and to trigger the RF generator *via* a frame grabber card after a target cell was detected. After background subtraction, a contour finding algorithm was applied^78^ to detect the convex hull of the contour. The cell size (*A*) is calculated from the projected cross-sectional area of the cell within the contour of the binarized image. Deformation (*D*) was derived from the perimeter (*l*) and the area (*A*) of the particle, using 
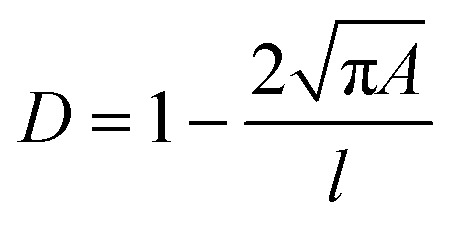
. Area ratio is the ratio of the area of the convex hull of the detected contour and the area of the original contour. The brightness of an object was obtained by masking the grayscale image using the original contour and calculating the mean of all grayscale values inside the contour.^79,80^

Experiments were performed using a total flow rate of 0.04 μL s^−1^ and 0.08 μL s^−1^, for a primary deformation channel of 20 μm and 30 μm respectively. The average cell velocity was ∼15 cm s^−1^ in both settings. The center of the measurement region (region of interest – ROI) was 60 μm away from the sorting region.

FEP tubing (0.0625 inch OD, 0.03 inch ID; no. 1520XL, Postnova Analytics) were used to connect the sheath and sample inlets of the soRT-DC chip to syringes (BD Luer-Lock 5 ml syringe no. 613-2043 P and BD Luer-Lock 1 ml syringe no. 613-4971; VWR) controlled *via* high precision syringe pumps (Nemesys S, Cetoni). A negative pressure controller (Lineup flow EZ™, Fluigent), operating in the range of 0–25 mbar, was connected to the default outlet. The negative pressure pump was adjusted to ensure that the cell stream was close to the bifurcation wall (Video S1†), which is necessary to translate the cell of interest into the target outlet upon FTSAW actuation. Target cells were collected into a 1.5 ml Eppendorf tube.

### Measurement buffer preparation

For all sorting experiments we used a high viscosity measurement buffer (MB) produced using 0.6% (w/w) methyl cellulose (4000 cPs; Alfa Aesar) diluted in phosphate buffer solution (PBS) without calcium and magnesium, adjusted to an osmolality of 310–315 mOsm kg^−1^ and pH 7.4. The viscosity of the buffer was adjusted to (25 ± 0.5) mPa s at 24 °C using a falling sphere viscometer (HAAKE Falling Ball Viscometer Type C, Thermo Fisher Scientific). The use of a high viscosity fluid for sorting experiments ensured slower cell sedimentation rate compared to PBS. Moreover, due to its high viscosity, the MB enhanced cell deformation due to hydrodynamic forces in a cell-comparable sized microfluidic channel.

### Polyacrylamide bead preparation

A mixture of polyacrylamide microgel beads of comparable elasticity and two different sizes was dispersed in 0.6% MB to achieve a final concentration of 8 × 10^6^ beads per ml. The beads were produced as described in a previous study.^53^ Briefly, a flow-focusing droplet microfluidic chip was used to produce droplets dispersed in an oil phase. The droplets had a well-defined diameter and contained a polyacrylamide pre-gel mixture with a total monomer concentration of 7.9%. After polymerization, the final beads were washed and stored in PBS. All bead sorting experiments were performed using a 30 μm microfluidic chip, a sheath flow rate of 0.07 μl s^−1^, and a sample flow rate of 0.01 μl s^−1^.

### Cell culture and preparation

Kc167 Drosophila cells (from B. Baum at the MRC Laboratory for Molecular Cell Biology, University College London, UK) were cultured in M3 shields and Sand medium (Invitrogen) supplemented with 10% heat-inactivated FBS (no. 10270106, Thermo Fisher Scientific) and 1% penicillin–streptavidin (no. 15140122, Thermo Fisher Scientific) under atmospheric CO_2_ concentration at 24 °C.

HL60/S4 cells were cultured in ATCC-modified RPMI-1640 medium (no. A1049101, Thermo Fisher Scientific) supplemented with 10% heat-inactivated FBS and 1% penicillin–streptavidin, at 37 °C, 5% CO_2_. The cells were passaged every 48 hours and re-suspended to a concentration of 1.2 × 10^6^ cells per ml. For the sorting experiments cells, were counted using a hemocytometer, then centrifuged at 200*g* for 3 minutes, and the cell pellet was resuspended in MB at the desired concentration; ranging from 3 × 10^6^ cells per ml to 36 × 10^6^ cells per ml. All sorting experiments for HL60/S4 cells were performed using a 30 μm microfluidic chip, a sheath flow rate of 0.07 μl s^−1^, and sample flow rate of 0.01 μl s^−1^.

HeLa cells (HeLa Kyoto) were cultured at 37 °C, 5% CO_2_, in high glucose DMEM (Dulbecco's modified Eagle medium; Thermo Fisher Scientific) supplemented with 10% foetal bovine serum (FBS; Thermo Fisher Scientific) and 100 units per ml penicillin/streptomycin (Thermo Fisher Scientific). Cells were sub-cultured at approximately 70% confluency. Briefly, the cell culture medium was aspirated, cells were washed with PBS and incubated with 0.25% trypsin/EDTA (Thermo Fisher Scientific) for 5 minutes at 37 °C. Detached cells were resuspended in culture medium, centrifuged at 200*g* for 3 minutes and counted using a haemocytometer. For sorting experiments, cells were seeded 48 hours prior to the experiments, at a density of 10 000 cells per cm^2^. After trypsinisation cells were counted using an automated cell counter (LUNA-II™; Logos Biosystems, South Korea) and resuspended in MB at a concentration of 3 × 10^6^ cells per ml. Prior to sorting HeLa were mixed at a 1 : 1 ratio with HL60/S4 cells also resuspended at 3 × 10^6^ cells per ml. Sorting experiments of a mixture of HeLa and HL60/S4 cells were performed using a 30 μm microfluidic chip, a sheath flow rate of 0.07 μl s^−1^ and sample flow rate of 0.01 μl s^−1^.

### Cell viability and proliferation assay

HL60/S4 cells resuspended in MB and exposed to TSAW during sorting were collected in the target outlet and used for monitoring cell proliferation and viability. For comparison, we included the following controls: 1) initial: cells resuspended in MB but not passed through the microfluidic channel; 2) default: cells collected during the sorting experiment in the default outlet, *i.e.* all non-target cells; 3) culture medium: cells that were never exposed to MB; 4) no SAW: cells that were resuspended in MB and passed through the microfluidic chip without activating the FTSAW. Briefly, following sorting 1 ml of medium was added to all cells suspended in MB and centrifuged at 200*g* for 5 minutes. The cell pellets were resuspended in 1.3 ml of culture medium and the total cell number was calculated using a haemocytometer and erythrosin B stain (Logos Biosystems). For each condition tested, equal number of cells were transferred in triplicates in a 48-well plate. The number of cells seeded was adjusted for each experiment according to the total number of cells in the target. The total number of cells and the viability was then measured daily using trypan blue staining (Merck KGaA; Sigma-Aldrich, Germany) and LUNA-II™ automated cell counter. After 48 hours the cells were transferred to a 12-well plate with 2 ml of fresh medium to provide enough nutrients and space for growth. Viability and proliferation were monitored for a total of 4 days.

### Blood preparation

Whole blood was drawn from human donors at Uniklinik Erlangen (Germany) after informed consent into 10 ml sodium-citrate tubes (S-Monovette 10 ml 9NC; no. 02.1067.001, Sarstedt). RBC-depleted blood was prepared using dextran-sedimentation. Briefly, 6% dextran (Dextran T500, Pharmacosmos) diluted in 0.9% NaCl (Sigma Aldrich) was added to whole blood at a 1 : 4 ratio, gently mixed by inverting and allowed to settle for 30 minutes at room temperature. Following sedimentation of RBCs, the upper layer that contains the plasma, was carefully transferred to a 50 ml falcon tube, filled with PBS solution. Cells were centrifuged at 100*g* for 10 minutes, the supernatant was removed, and the cell pellet was resuspended in 200 μl of 0.6% MB for sorting experiments. For whole blood measurements, the blood was diluted in a 1 : 50 dilution in the MB. WBCs, platelets and neutrophils were sorted using a 30 μm microfluidic chip, a sheath flow of 0.07 μl s^−1^ and a sample flow of 0.01 μl s^−1^. Lymphocytes and myeloid cells were sorted using a 20 μm microfluidic chip, a sheath flow of 0.03 μL s^−1^ and a sample flow of 0.01 μl s^−1^.

### Data analysis

The data analysis was processed using Shape-Out version 2.7.3 (available at https://github.com/ZELLMECHANIK-DRESDEN/ShapeOut2), Python 3.7 and Origin Pro 2020. Table 1 describes in detail the sorting gating strategies and the filtering gates used for plotting and calculating the purity.

## Conclusion

In this work, we showed how the integration of FIDT into a standard RT-DC setup can enable sorting by FTSAW with a throughput, stability, flexibility, and purity superior to other image-based sorting methods. High frequency (135 MHz) FIDTs produce concentrated acoustic energy within a 50 μm region such that a 1 ms pulse is enough to translate the target cell moving in a high viscous buffer into the desired outlet at a throughput of up to 400 cells per s. Our experimental results demonstrated the applicability of the soRT-DC method to different cell types in a wide range of sizes and mechanical properties resembling most mammalian cells of interest for biological and biophysical applications. We have shown that the use of FTSAW instead of SSAW eliminates the use of the tedious pressure-node setting step. Furthermore, the new chip geometry allows for long sorting experiments to enrich small cell fraction within a concentrated heterogeneous population. Overall, our FTSAW based soRT-DC setup provides a user-friendly, label-free, reliable, and high-throughput device for continuous cell sorting. This can enable sorting of different cell types based on multiple physical parameters with high purity and sensitivity. This is essential for downstream biological applications, such as – omics studies, drug screening or transplantation,^28^ where a large number of cells is required. Finally, we demonstrate a new gating strategy to sort cells based on their mechanical properties. We envision that in the future this will provide a unique approach to investigate how intrinsic or induced variation in cell mechanical properties can dictate cell function which could be harnessed for novel therapeutic approaches. The FTSAW actuator itself, thanks to its flexibility, robustness, easy operation and integration, represents a valuable option for sorting integration of forthcoming technologies. In the future, FIDT with smaller aperture and higher/multiple frequencies can be designed and tested to further reduce the gap between sorting rate and cell analysis throughput.

## Author contributions

Conceptualization: A. A. N., S. G. and J. G.; methodology: A. A. N., D. S., R. G., M. H., S. G. and J. G.; investigation: A. A. N., D. S., C. K. X. and S. G.; data analysis and interpretation: A. A. N., D. S., C. K. X., and S. G.; writing – original draft: A. A. N., D. S. and S. G.; writing – review & editing: A. A. N., D. S., C. K. X., R. G., M. H., S. G. and J. G.; funding acquisition: S. G. and J. G.; supervision: S. G. and J. G.

## Conflicts of interest

The authors declare that they have no competing interests.

## Supplementary Material

LC-023-D2LC00636G-s001

LC-023-D2LC00636G-s002
