# Supplementary material for: Image-based cell sorting using focused travelling surface acoustic waves
Source: Lab Chip. 2023 Jan 9;23(2):372–87. doi: 10.1039/d2lc00636g (PMC9844123; doi:10.1039/d2lc00636g)
Supplement: LC-023-D2LC00636G-s001 [file LC-023-D2LC00636G-s001.pdf]

Electronic Supplementary Material (ESI) for Lab on a Chip.
This journal is © The Royal Society of Chemistry 2022

Image-based cell sorting using focused travelling surface acoustic waves

Ahmad Ahsan Nawaz[1*], Despina Soteriou[1*], Catherine K Xu[1], Ruchi Goswami[1], Maik Herbig[2], Jochen Guck[1], Salvatore Girardo[1]

[1]Max Planck Institute for the Science of Light & Max-Planck-Zentrum für Physik und Medizin, Erlangen, Germany

[2]Department of Chemistry, University of Tokyo, Tokyo, Japan.

*These authors contributed equally

Supplementary Information

Video S1: Fine adjustment of the pressure value at the default outlet to enable cell sorting upon actuation. Cells are seen flowing into the default outlet when the pressure controller is set at -25 mBar. When the pressure is adjusted to -5.7 mBar the cell stream comes closer to the bifurcation wall which is necessary to translate the cell of interest into the target outlet upon FTSAW actuation.

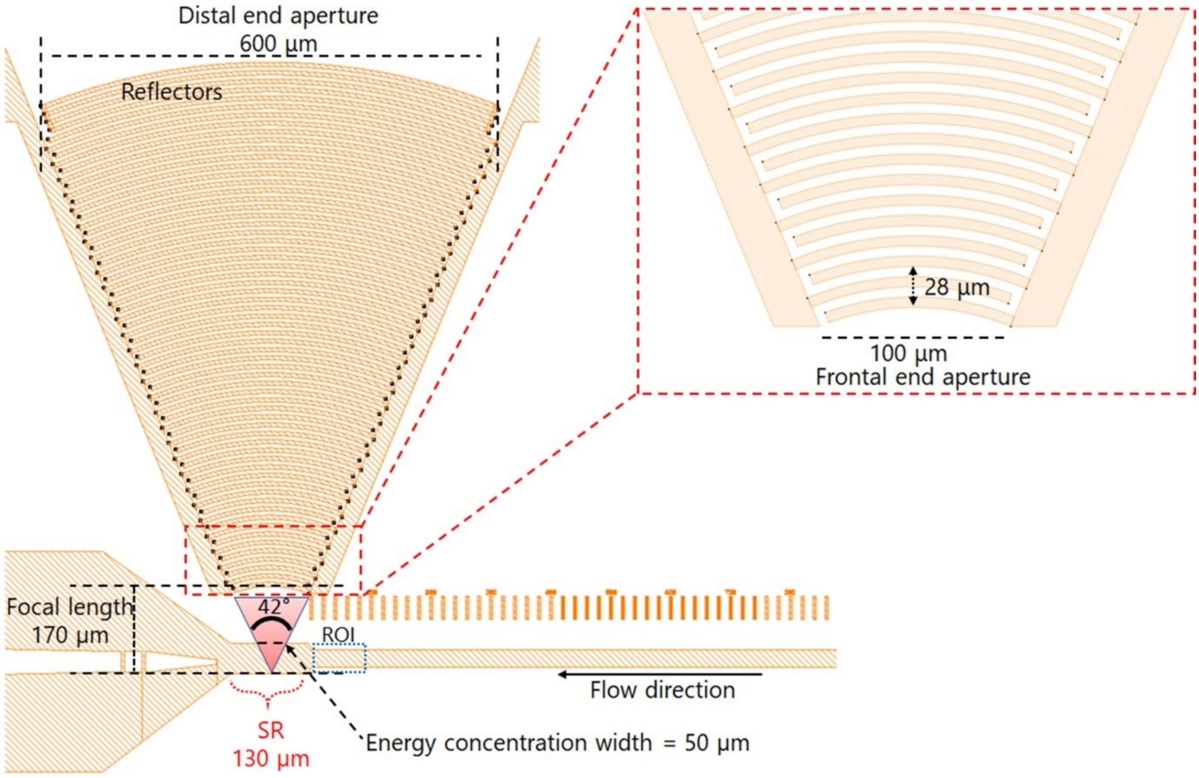

Figure S1: FIDT design. Schematic overview (Klayout software) of the FIDT aligned in front of the sorting region (SR length=130µm) in a soRT-DC chip. The FIDT has a distal end aperture of 600 µm and a frontal end aperture of 100 µm subtending an angle of 42°. This provides a focal point located close to the SR channel wall at 170 µm from the first FIDT finger. The red-shaded triangular region depicts the energy concentration within a region of 50 µm in the sorting region. The inset shows a FIDT with periodicity of 28 µm. The center of the region of interest (ROI) is located 60µm from the SR.

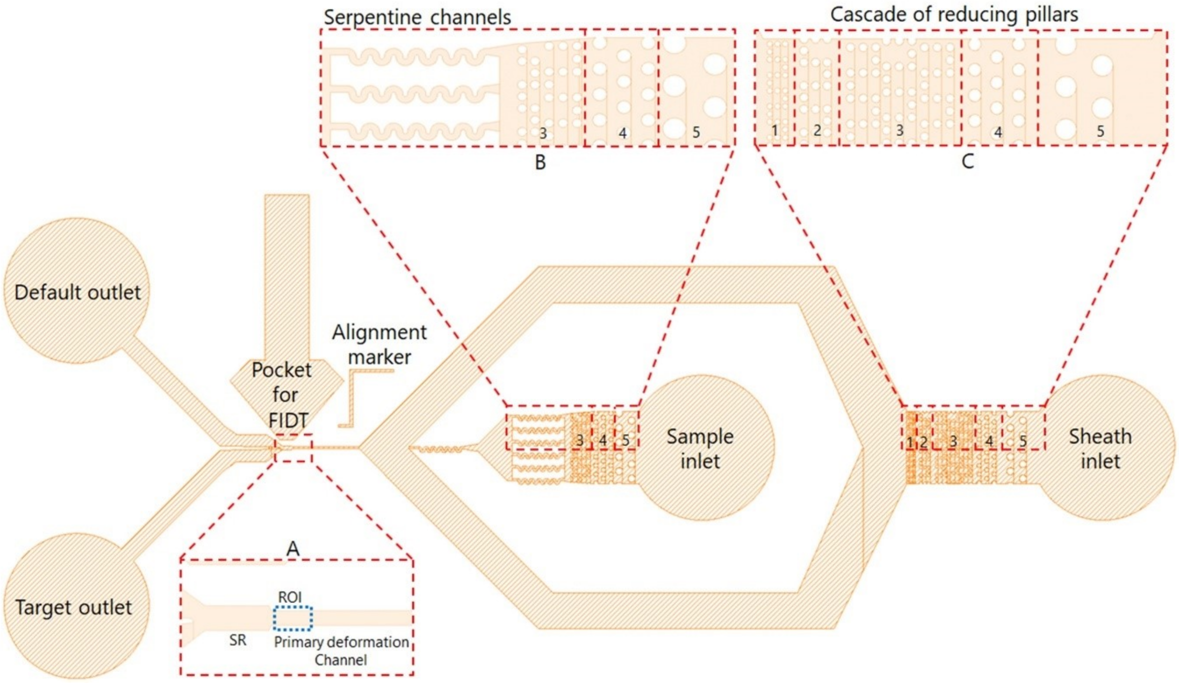

Figure S2: Chip design. Schematic overview (Klayout Software) of the PDMS channel geometries of the soRT-DC chip depicting inlets, outlets, pocket for FIDT, alignment marker and filter architectures. Bottom inset shows an expanded view of the sorting region and the primary deformation channel. Top insets show an expanded view of the filter architecture at the sheath and sample inlets. Region 1: pillar diameter=25 µm and inter-pillar distance=10 µm; Region 2: pillar diameter=40 µm and inter-pillar distance=15 µm; Region 3: pillar diameter=40 µm and inter-pillar distance=20 µm; Region 4: pillar diameter=60 µm and inter-pillar distance=60 µm; Region 5: pillar diameter=100 µm and inter-pillar distance=100 µm. Top left inset shows the serpentine-like channels for breaking apart cell clumps.

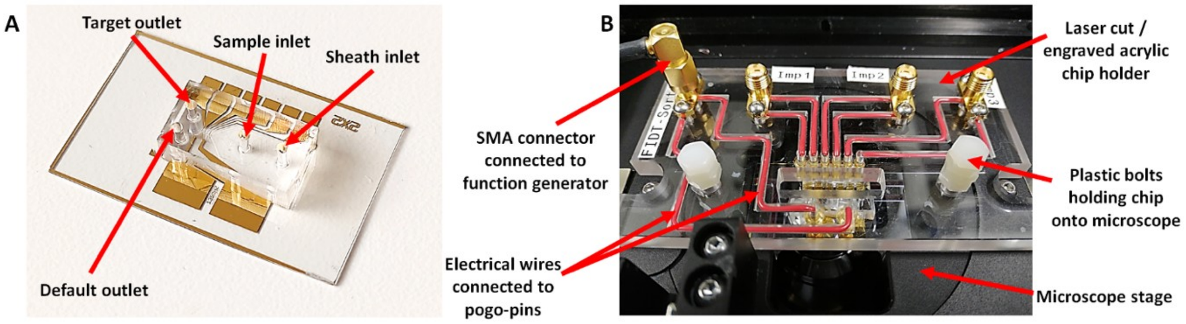

Figure S3: soRT-DC chip and chip holder. (**A**) picture of the assembled soRT-DC chip. Bottom layer: LiNbO3 substrate integrating the FIDT. Top layer: microstructured PDMS element including the microfluidic chip geometry. (**B**) chip holder for mounting the chip on the microscope stage holder and for interfacing it with the external RF generator by the SMA connector.

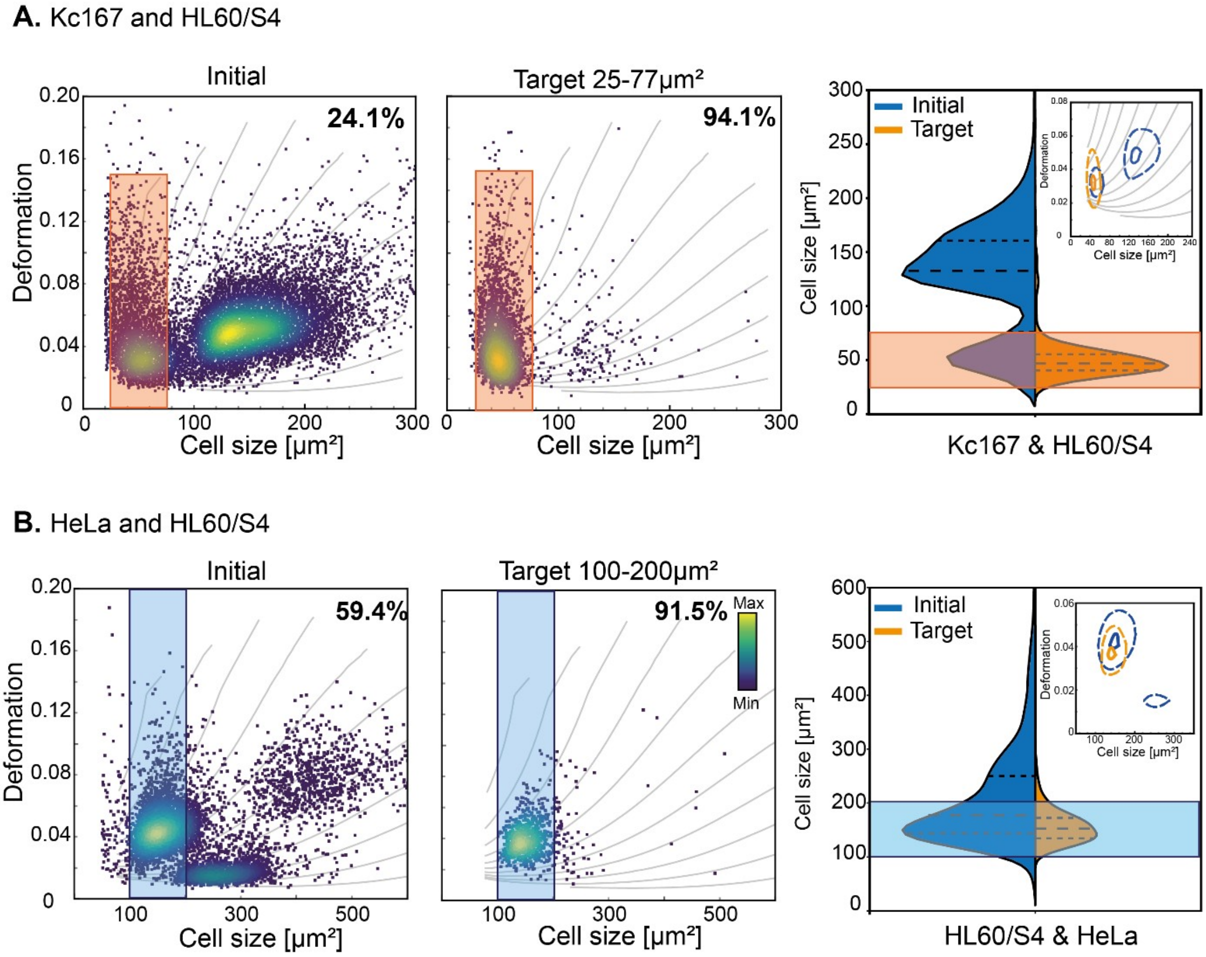

Figure S4: Size-based sorting of a mixture of two cell lines (**A**) Deformation vs cell size scatter plot of a mixture of Kc167 (smaller size) and HL60/S4 (bigger size) cells mixed at a 1:4 ratio. The left scatter plot represents the initial populations and the middle scatter plot the sorted target cell population with a 4-fold enrichment from 24.1% to 94.1%. The orange shaded rectangle represents the sorting gate (25–77 μm^2). (**B**) Deformation vs cell size scatter plot of a mixture of HL60/S4 (small cells) and HeLa (big cells) mixed in 1:1 ratio. The left scatter plot represents the initial populations and the middle scatter plot the sorted target cell population with a 1.5-fold enrichment from 59.4% to 91.5%. The blue shaded rectangle represents the sorting gate (100–200 μm^2). The colour map in scatter plots represents the event density. The violin plots show the distribution of the corresponding scatter plot data for the initial and target cell populations. The percentages on the scatter plots indicate the fraction of cells in the sorting gate. The insets show merge contour plots of the initial and target populations; 95%-density (dashed lines) and 50%-density (solid lines).

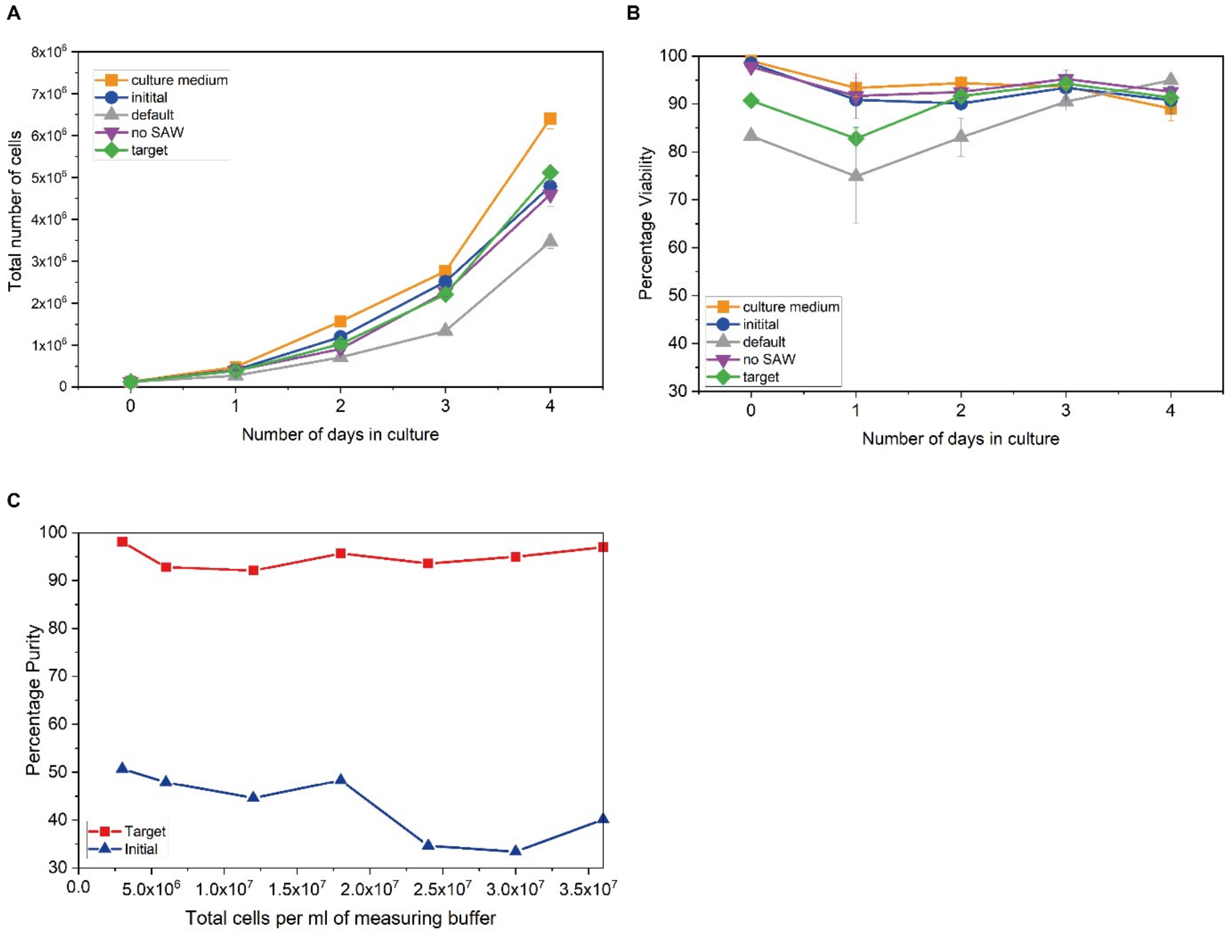

Figure S5: Assessing proliferation, viability, and purity of cells after sorting. (**A**) Proliferation and (**B**) Viability of HL60/S4 cells after exposure to FTSAW. Target HL60/S4 cells (Target; green) were cultured post-sorting for 4 days and compared to: *i*) cells that were not exposed to TSAW (no SAW; magenta); *ii*) cells that were diluted in measuring buffer (Initial; blue); *iii*) cells that were loaded on the microfluidic chip but were not sorted (Default; grey); and *iv*) control cells (culture medium; orange). Viability was assessed by counting the number of live and dead cells at each time point. (**C**) Percentage purity of initial (blue) and target (red) HL60/S4 cells diluted in MB with increasing concentration. The target purity (TP) is defined as the percentage ratio of the number of selected cells to the total number of cells in the sorted-target; and the initial purity (IP) as the percentage ratio of the number of selected cells to the total number of cells in the unsorted-initial population.

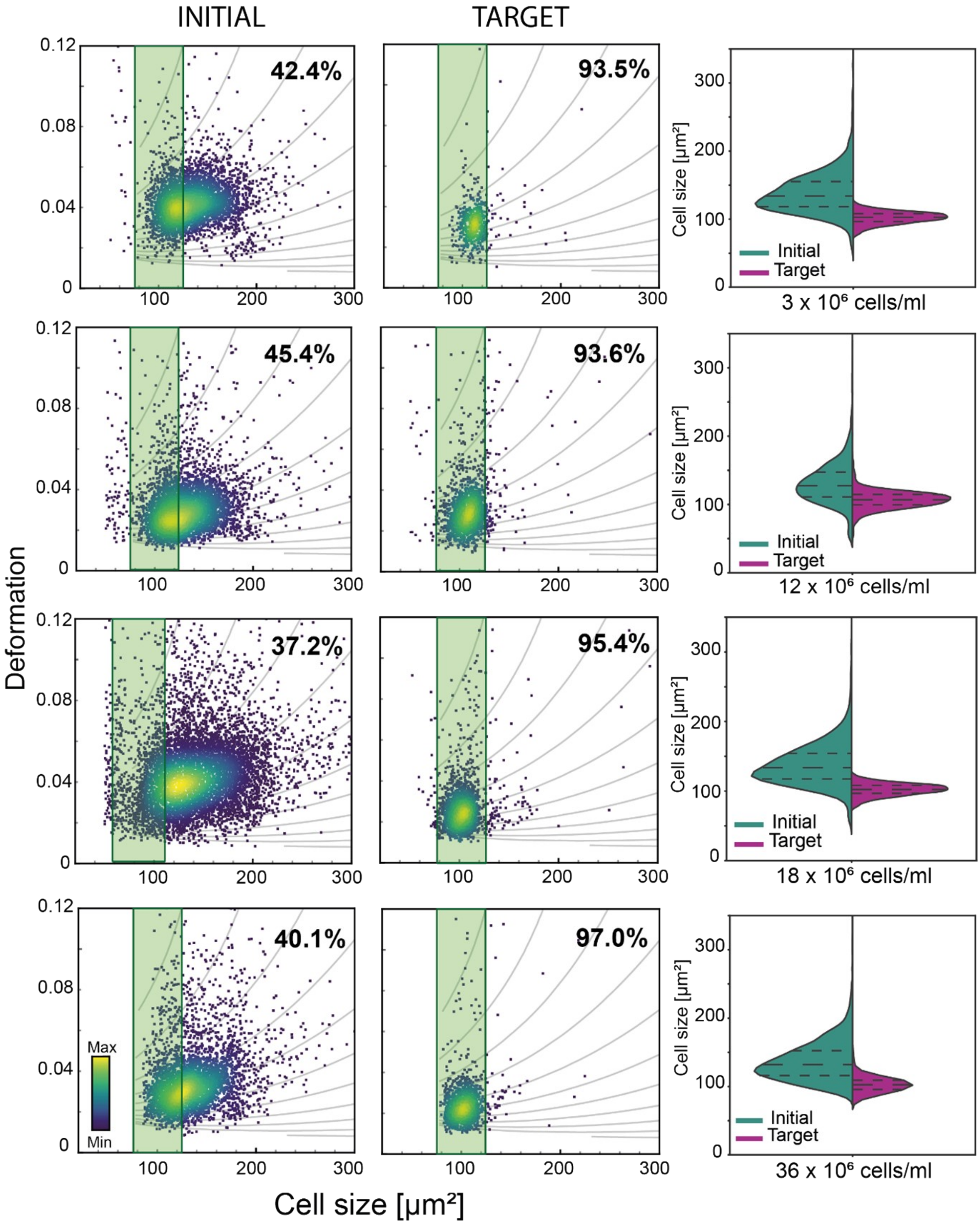

Figure S6: Sorting reproducibility with increasing concentration of cells. Representative scatter plots of deformation vs cell size scatter plots of HL60/S4 diluted at a different concentration in MB (3×10^6, 12×10^6, 18×10^6 and 36×10^6cells/ml). The colour map in scatter plots represents the event density. The violin plots show the distribution for both the initial and the target cell populations. The percentages on the scatter plots indicate the fraction of cells in the sorting gates.
